# Graphene and Other 2D Layered Nanomaterials and Hybrid Structures: Synthesis, Properties and Applications

**DOI:** 10.3390/ma14237108

**Published:** 2021-11-23

**Authors:** Domenica Scarano, Federico Cesano

**Affiliations:** Department of Chemistry and NIS Interdepartmental Centre, University of Torino, Via P. Giuria 7, 10125 Torino, Italy; domenica.scarano@unito.it

The field of two-dimensional (2D) layered nanomaterials, their hybrid structures, and composite materials has been suddenly increasing since 2004, when graphene—almost certainly the most known 2D material—was successfully obtained from graphite via mechanical exfoliation [1]. Since then, 2D crystals and layered nanomaterials have been more actively and widely involved in research, as shown by the growing number of scientific contributions (Figure 1a). From a geographical point of view, the majority of such contributions are from China (c.a. 29%) and the United States (18%), followed by South Korea, Germany and India (4%), United Kingdom, Singapore and Japan (3%). These scientific contributions categorized by subject area suggest a clear direction towards the application of these materials in various sectors, including engineering, energy, biochemistry-genetics-molecular biology, and computer science (Figure 1b). In this regard, there are actually emerging applications in many fields, including electronics, sensing, spintronics, plasmonics, photodetectors, ultrafast lasers, batteries, supercapacitors, piezoelectrics, thermoelectrics and catalytic applications [2,3,4,5,6]. Such applications are the results of the extremely wide variety of 2D materials that have been fabricated and studied, including inorganic, organic, hybrid compounds and heterostructures according to a traditional definition (Figure 2). Hundreds of compounds potentially stable at the atom-thin layer have been identified [7] and expand the actual group of 2D materials. As for the 2D material family, graphene is undoubtedly the most studied material, followed by MoS_2_, which represents the transition metal dichalcogenide compounds (MX_2_ type, in which M is a transition metal, such as Mo or W and X a chalcogen atom, such as S, Se, or Te). Single-element 2D materials (i.e., borophene, phosphorene, etc.), with rare exceptions, redefine the physics and chemistry of the elements [8], while ternary 2D materials have additional freedom degrees for tailoring their band gaps and physicochemical properties via stoichiometric engineering [9].

The assembly of 2D materials directly onto the surface of solids (such as in-situ fabrication of photosensitizers at TiO_2_ surface [10]) is still an evolving field, but the engineered interface can improve some properties with respect to heterostructures with weakly bonded van der Waals interactions.

As reported in the previous special issue [11], the current one highlights a few achievements, past/present developments, and future perspectives in the 2D layered nanomaterials and the related hybrid structures fields. It includes two reviews and height research articles. 

Yu et al. [12] reviewed the subject of graphene-based materials (i.e., graphene, graphene oxide or reduced graphene oxide) as emerging new electrode material in electro-Fenton reactions to be adopted in the contaminant removal from water, thus preserving valuable water resources. Notably, when graphene or graphene analogues are combined and supported with other carbon materials, such as carbon fiber felts or CNTs, and with Fe or other metal oxide catalysts have the potential to provide true Fe- and metal-free E-Fenton catalysts. For instance, reduced-graphene oxide may be on carbon felts or graphite electrodes and combined with CNTs, to be used in gas diffusion electrodes and when doped with N and other elements, the N-doping is appearing to be the best option in E-Fenton.

In another review, Alves et al. [13] reviewed the field of graphene and graphene derivatives to be employed with bio-adsorbents. Among all, GO combined with chitosan has the potential to remove organic pollutants and metal cations that escaped into the water environment. The mutual role played by graphene (mechanical properties) and chitosan hydrogel (i.e., immobilization matrix) of the composite materials can increase sorption capabilities and performances as compared to graphene or chitosan alone as independent sorbent materials. The authors have also shown that additional components (including magnetic iron oxides, chelating agents, cyclodextrins, additional adsorbents and polymeric blends) can be positively added. In this regard, the performances of these materials in the removal of organic molecules, dyes and heavy metal ions are discussed together with regeneration strategies, selectivity in the adsorption process and involved costs.

The topic of graphene synthesis is still relevant for the exploration of more favorable conditions and for mass graphene production. Tan et al. [14] reported a method to obtain few-layer graphene under semi-open environmental conditions by introducing arc-discharge plasma technology. Compared with other fabrication technologies (i.e., chemical vapor deposition, mechanical/chemical exfoliation and chemical reduction of GO), no toxic gases and hazardous chemicals are generated. In the plasma discharge process, when the gas between the cathode and the anode is activated by the arc-discharge, a hexagonal arrangement of carbon atoms is observed, resulting from the nucleation and rearrangement of individual carbon atoms that have evaporated at the anode and cathode. Gudaitis et al. [15] demonstrated that graphene can be grown on the Si (100) substrates. More in detail, few-layer graphene was synthesized from CH_4_ and H_2_ on the Si (100) substrates without the use of catalysts via the use of direct microwave plasma-enhanced chemical vapor deposition (PECVD). N-type self-doping graphene is obtained in the process, due to the charge transfer from the Si (100) substrate. The authors observed large graphene sheets with the occurrence of compressive stresses, presumably arising from thermal stress due to the huge lattice mismatch between the Si (100) substrate and the growing graphene.

The theme of graphene functionalization/dispersion is still very active, particularly for environmentally friendly and effective methods that do not require the use of strong acids or oxidants. Farivar et al. [16] reported a simple and green modification method for preparing highly dispersible functionalized graphene via thermal thiol-ene click reactions. The method provides specific chemical functionalities such as –COO, –NH_2_ and –S to the graphene sheets by using modified L-cysteine ethyl ester. The direct attachment of specific functional groups on the surface of graphene is obviously highly demanded towards the application of such materials, including ink formulations, coatings, adsorbents, sensors and supercapacitors.

Poyato et al. [17] reported the preparation of graphene nanosheets via the electrochemical exfoliation method and fabricated Yttria tetragonal zirconia (3YTZP)-based composites. The authors investigated the morphology, structure and surface properties of the composites, which were found to be formed of a graphene layer with stacking number n < 10, containing amorphous carbon and vacancy-type defects. Finally, the authors verified their Vickers hardness, which was compared with that of sintered monolithic 3YTZP ceramics. As for defectivity, there is a growing number of theoretical reports highlighting the role played by the different types of structural defects and properties.

Along this research line, Slepchenkov et al. [18] investigated a method for controlling the electronic properties of nanoporous carbon glass-like surfaces, when the pores are filled with K atoms. The presence of surface impurities, such as chemically adsorbed H and O atoms, and -OH groups, was investigated. The authors showed the calculated work function in the presence of impurities on the carbon nanoporous surface. Furthermore, the state of K atoms was shown, providing insights for the effective control of the properties, such as electronic structure and emission.

Xu et al. [19] reported the growth of monolayer WS_2_ on SiO_2_ substrate via the chemical vapor deposition (CVD) process in the presence of ZnO crystalline whiskers as growth promoters. As monolayer WS_2_ was found on both sides of ZnO crystal whiskers, the authors discussed the monolayer growth mechanisms by approaching a concentration distribution model. According to this growth model, S and W volatile compounds and their concentrations were suggested to play a role in the WS_2_ sheet thickness. Arevalo et al. [20] have recently shown the preparation of heterostructures formed by thin borocarbonitride (BCN) layers grown on TiO_2_ nanoribbons. Such nanoribbons were first obtained by thermal oxidation of TiS_3_ samples. Then, BCN layers were successfully grown by PECVD. The obtained TiO_2_-BCN heterostructures were successfully employed in a photoelectrochemical cell, showing a boosted current density under dark conditions and higher photocurrents when compared with the bare TiO_2_. The excellent photo-electrocatalytic properties of BCN suggest its use as a metal-free material in water-splitting devices.

Drici-Setti et al. [21] prepared layered double hydroxides (LDHs) based on Co, Fe and acetate ions by forced hydrolysis in a polyol medium. The synthesized Co-Fe-acetate LDH exhibited anion exchange properties and the acetate interlayer species were successfully exchanged by carbonate anions with a topotactic reaction. The exchange reactions are also favored by the high interlamellar distance. The authors have also tested LDH-Co-Fe-acetate system in sorption experiments of azoic anionic dyes from wastewater and high dye uptake was observed due to both physisorption and chemical sorption processes.

We truly hope that the reviews and research articles collected in this special issue may benefit readers and researchers in diverse fields for rising their knowledge in the fields of 2D layered nanomaterials and of the related hybrid structures, thus motivating and giving motivation for new relevant studies. We also express our sincere gratitude toward the authors, referees and the editorial staff for their valuable contributions, appropriate and insightful comments, and for the rapid and constant support.

## Figures and Tables

**Figure 1:**
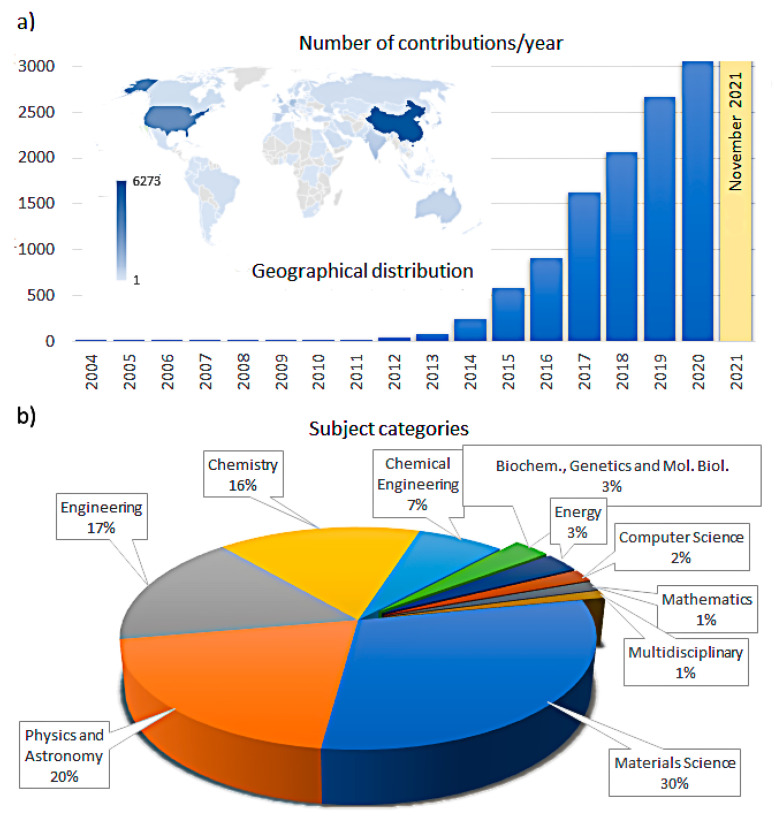
Scientific contributions dedicated to 2D materials (**a**) numbers of document distributed in the last 20 years and their geographical distribution; (**b**) subject areas. Keyword: “2D Materials” (source: Scopus).

**Figure 2:**
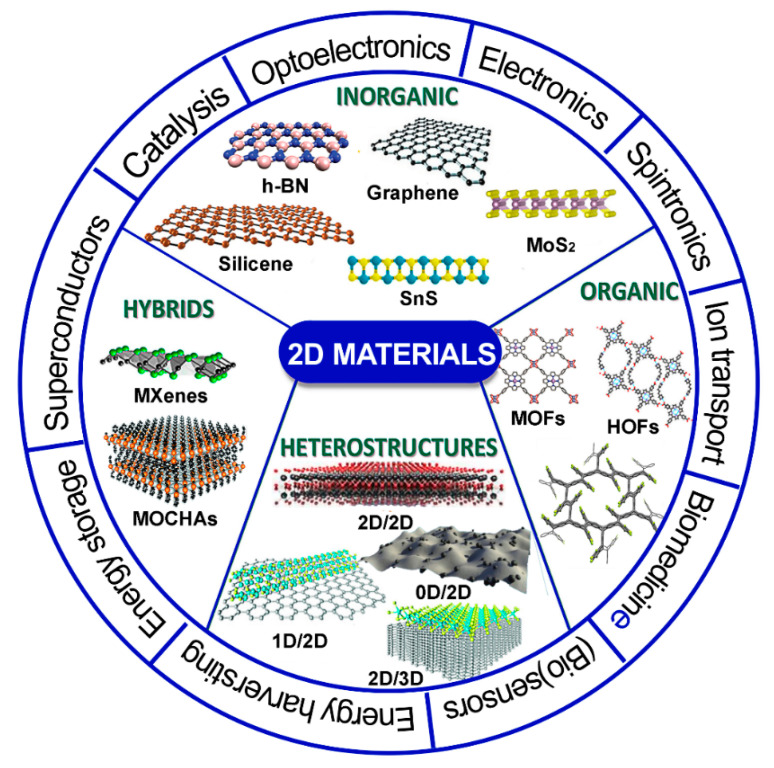
Classification of 2D materials and their most relevant applications.
